# Heterogeneous Graph Convolutional Networks and Matrix Completion for miRNA-Disease Association Prediction

**DOI:** 10.3389/fbioe.2020.00901

**Published:** 2020-08-20

**Authors:** Rongxiang Zhu, Chaojie Ji, Yingying Wang, Yunpeng Cai, Hongyan Wu

**Affiliations:** ^1^Joint Engineering Research Center for Health Big Data Intelligent Analysis Technology, Shenzhen Institutes of Advanced Technology, Chinese Academy of Sciences, Shenzhen, China; ^2^Shenzhen College of Advanced Technology, University of Chinese Academy of Sciences, Shenzhen, China; ^3^Department of Neurology and Stroke Center, The First Affiliated Hospital of Jinan University, Guangzhou, China; ^4^Clinical Neuroscience Institute, The First Affiliated Hospital of Jinan University, Guangzhou, China

**Keywords:** miRNA disease, matrix completion, GCNs, heterogeneous graph, association prediction

## Abstract

Due to the cost and complexity of biological experiments, many computational methods have been proposed to predict potential miRNA-disease associations by utilizing known miRNA-disease associations and other related information. However, there are some challenges for these computational methods. First, the relationships between miRNAs and diseases are complex. The computational network should consider the local and global influence of neighborhoods from the network. Furthermore, predicting disease-related miRNAs without any known associations is also very important. This study presents a new computational method that constructs a heterogeneous network composed of a miRNA similarity network, disease similarity network, and known miRNA-disease association network. The miRNA similarity considers the miRNAs and their possible families and clusters. The information of each node in heterogeneous network is obtained by aggregating neighborhood information with graph convolutional networks (GCNs), which can pass the information of a node to its intermediate and distant neighbors. Disease-related miRNAs with no known associations can be predicted with the reconstructed heterogeneous matrix. We apply 5-fold cross-validation, leave-one-disease-out cross-validation, and global and local leave-one-out cross-validation to evaluate our method. The corresponding areas under the curves (AUCs) are 0.9616, 0.9946, 0.9656, and 0.9532, confirming that our approach significantly outperforms the state-of-the-art methods. Case studies show that this approach can effectively predict new diseases without any known miRNAs.

## 1. Introduction

MicroRNAs (miRNAs) are a class of short non-coding single-stranded RNA molecules (22 nt) encoded by endogenous genes (Ambros, 2001). Studies have shown that miRNAs are involved in the emergence and development of various human diseases (Alvarez-Garcia and Miska, 2005; Jopling et al., 2005). Therefore, finding the associations between miRNAs and diseases could contribute to pathological classifications, individualized diagnoses, and disease treatments. However, experimental methods for identifying associations between miRNAs and diseases are expensive and time-consuming. Therefore, computational methods have drawn wide attention to reveal potential associations between miRNAs and diseases.

Based on the known miRNA-disease associations, a number of computational methods have been proposed to predict candidate miRNAs for diseases. These methods cover three main categories: network algorithms, machine learning, and matrix-based methods.

Jiang et al. (2010) proposed the first computational method, which integrated a miRNA functional similarity network, disease phenotype similarity, known disease-miRNA association network and discrete probability distribution named the hypergeometric distribution to predict the potential associations. Xuan et al. (2013) developed a model named HDMP. The miRNA functional similarity was calculated according to disease terms and the disease phenotype similarity. HDMP could not predict candidate miRNAs for new diseases without any known associated miRNAs, however. Both methods considered only local neighbor similarity information of each miRNA, so they did not achieve satisfactory performance. To make full use of network information, Chen et al. (2012) developed the global network method RWRMDA that implemented random walks on a miRNA functional similarity network. However, this model could not address new diseases associated with no miRNAs. Many other models have incorporated complex interaction networks to present the relationship between miRNA and disease. For example, Mørk et al. (2014) proposed a model of miRNA-protein-disease (miRPD) association prediction with proteins as mediators. The authors verified the associations between miRNAs and diseases by integrating both miRNA-protein and protein-disease associations.

Recently, some machine-learning-based models were also developed to predict potential miRNA-disease associations. Based on the K-nearest-neighbor approach for miRNAs and diseases, RKNNMDA (Chen et al., 2017) was used to rank K-nearest neighbors with SVMs and utilized weighted voting for each predicted miRNA-disease association. Zhao et al. (2019) developed a novel model of adaptive boosting for miRNA-disease association prediction (ABMDA). They used a decision tree as a weak classifier and combined weak classifiers, which could score samples to form a strong classifier based on corresponding weights.

Based on the information of known miRNA-disease associations and the similarity matrix, an inductive matrix completion algorithm was used to complete the missing entries of a known miRNA-disease association matrix. Li et al. (2017) released a method of matrix completion for an miRNA-disease association prediction model (MCMDA), which updated the adjacency matrix of known miRNA-disease association networks using matrix completion algorithms. Chen et al. (2018) also developed a model of inductive matrix completion for miRNA-disease association prediction (IMCMDA).

The methods of the three categories mentioned above have their own strengths and limitations. Combining the network algorithm, machine learning and matrix completion, we developed a matrix completion method based on graph convolutional networks for miRNA-disease association prediction. First, we constructed a heterogeneous network by integrating the miRNA similarity network, disease similarity network and known miRNA-disease associations. Inspired by Wan et al. (2019), we then obtained new node embedding by aggregating neighborhood information derived from the heterogeneous network based on graph convolutional operations, which can pass the information of a node to its intermediate and distant neighbors. To the largest extent, to preserve the topological information of the heterogeneous network, the loss function of reconstructing the entire heterogeneous network (matrix) was minimized during the training process. Finally, by comparing the reconstructed and original matrices, we discovered novel miRNA-disease associations. To evaluate the effectiveness of the proposed method, we implemented 5-fold cross-validation, leave-one-disease-out cross-validation (LODOCV), and global and local leave-one-out cross-validation (LOOCV) and obtained AUCs of 0.9616, 0.9946, 0.9656, and 0.9532, respectively. Furthermore, two types of case studies were carried out. As a result, most of the predicted miRNAs were confirmed by related databases. In conclusion, the proposed method can effectively predict potential miRNA-disease associations.

## 2. Materials and Methods

### 2.1. MiRNA-Disease Network

To construct the known miRNA-disease network, we downloaded the verified miRNA-disease associations from the HMDD database (Li et al., 2014). We used an adjacency matrix ***RD*** to describe the network. The element ***RD***(*i, j*) is 1 if miRNA *m*_*i*_ is associated with disease *d*_*j*_ and 0 otherwise. We obtained 6,441 associations between 577 miRNAs and 336 diseases after duplicates were removed.

### 2.2. Disease Functional Similarity Network

Similar diseases have a great probability of being regulated by similar genes. Therefore, we constructed a disease similarity network based on the gene functional information. The data can be downloaded from the HumanNet database (Li et al., 2011), which contains an associated log-likelihood score (LLS) of each interaction between two genes or gene sets. The similarity ***DS***(*i, j*) between diseases *d*_*i*_ and *d*_*j*_ can be calculated as follows:

(1) $$DS(i,j)=\{\begin{array}{c} \frac{\sum_{x\in S(d_{i}))}LLS(x,S(d_{j}))+\sum_{y\in S(d_{j}))}LLS(y,S(d_{i}))}{\left|S(d_{i}))|+|S(d_{j}))\right|},\\ 0, \end{array}+\begin{array}{c} \left|S(d_{i}))\right|\\ |S(d_{j}))|\neq 0\\ otherwise \end{array}$$

where *S*(*d*_*i*_) represents the gene sets related to disease *d*_*i*_; |*S*(*d*_*i*_)| represents the cardinalities of *S*(*d*_*i*_); and *LLS*(*x, S*(*d*_*j*_)) is the LLS between gene *x* and gene set *S*(*d*_*j*_).

### 2.3. MiRNA Similarity Network

MiRNA families feature a common sequence or structure configuration in sets of genes (Kaczkowski et al., 2009). The miRNA cluster is a set of two or more miRNAs that are transcribed from physically adjacent miRNA genes. MiRNAs belonging to the same family or cluster are expected to have similar functions and thus be associated with the same diseases. Therefore, we constructed a miRNA similarity network by combining verified miRNA-target associations, family information, cluster information, and verified miRNA-disease associations. In this process, first, the verified miRNA-target associations is downloaded from miRTarBase (Hsu et al., 2014). Two miRNAs are connected if they share common targets. The element value of ***RST*** (miRNA similarity based on target) represents the number of shared targets between miRNAs. Then, we can obtain the family information of miRNAs from miRBase (Griffiths-Jones et al., 2003). If two miRNAs belong to the same miRNA family, we set their ***RSF*** (miRNA similarity based on family) value to 1; otherwise, we set it to 0. Third, the miRNA cluster information is accessible in miRBase (Kozomara and Griffiths-Jones, 2014). If two miRNAs belong to the same cluster, then the ***RSC*** (miRNA similarity based on cluster) value is set to 1. Finally, we utilize MISIM, a miRNA similarity network based on verified miRNA-disease associations, to define ***RSD*** (miRNA similarity based on disease). Once the data are prepared, we combine the four matrices to calculate the similarity ***RS***(*i, j*) between miRNA *r*_*i*_ and miRNA *r*_*j*_:

(2) $$\begin{array}{r} RS\left(i,j\right)=\alpha \cdot RST\left(i,j\right)+\beta \cdot RSF\left(i,j\right)+\gamma \cdot RSC\left(i,j\right)+\theta \cdot RSD\left(i,j\right) \end{array}$$

where α = 0.2, β = 0.1, γ = 0.2, and θ = 0.5 are described as in the work (Zeng et al., 2018).

### 2.4. Heterogeneous Graph Convolutional Networks

#### 2.4.1. Heterogeneous Network Construction

**Figure 1:**
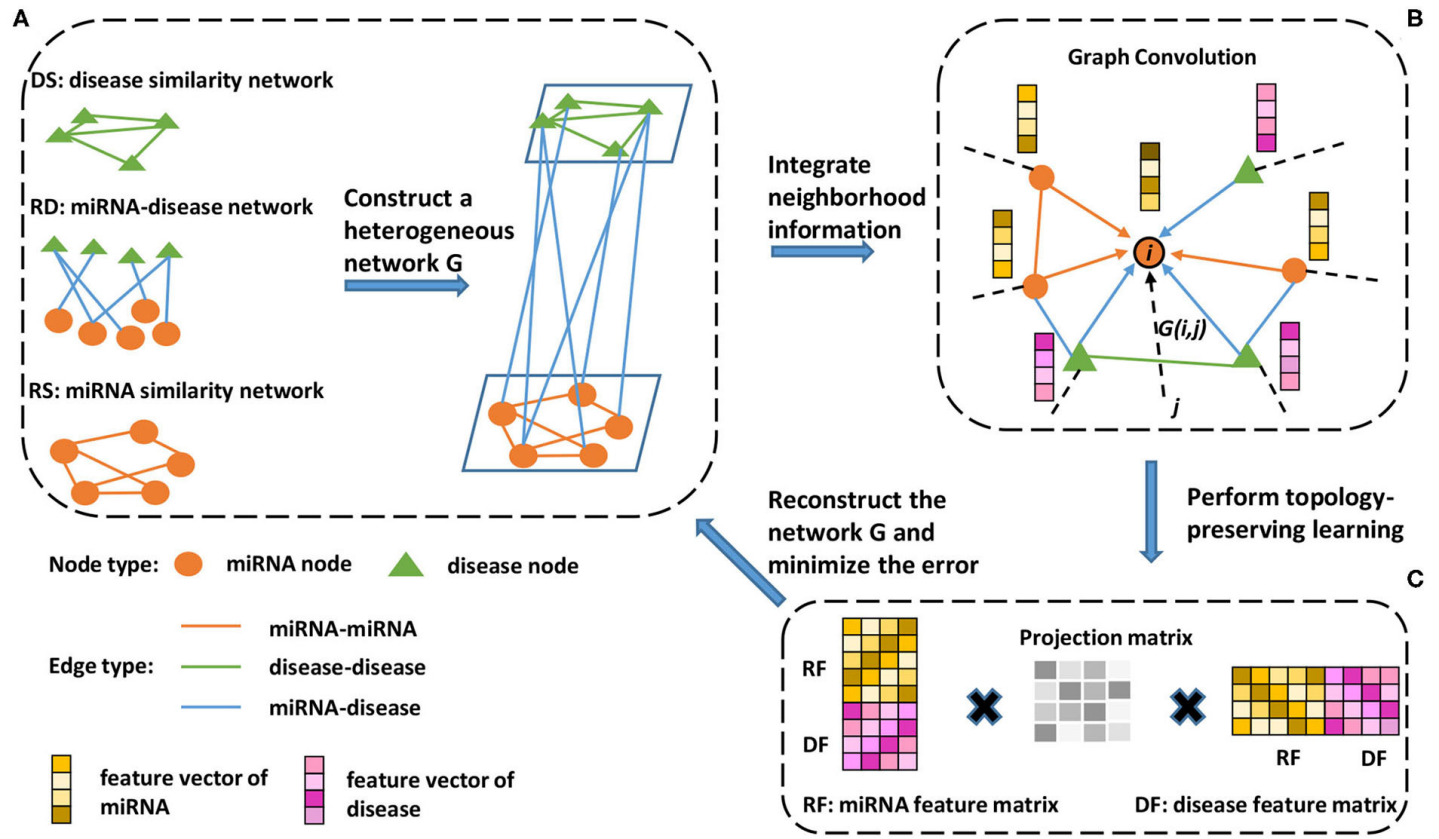
Schematic workflow of the proposed method. **(A)** A miRNA similarity network, disease similarity network, and miRNA-disease association network are used to construct a heterogeneous network. **(B)** To extract information from the neighborhood, a neighborhood information aggregation operation (Formula 5) is applied on every node. Then, each node updates its feature representation by concatenating its current representation with the aggregated information (Formula 6). **(C)** A feature matrix is constructed, each row of which is a new node feature representation (Formula 7). Then, the feature matrix is used to reconstruct the heterogeneous network, and topology-preserving learning is implemented by minimizing the reconstructed error (Formula 8).

As shown in Figure 1, we constructed a heterogeneous network based on the miRNA similarity network ***RS***, disease similarity network ***DS***, and miRNA-disease network ***RD***. The heterogeneous network can be represented as follows:

(3) $$\begin{array}{r} G=\left(N,E\right)=\left(\begin{array}{cc} RS & RD\\ RD^{T} & DS \end{array}\right) \end{array}$$

where N is the node set that contains two kinds of nodes NT = {miRNA, disease} , and E is the edge set ET = {miRNA-miRNA, miRNA-disease, disease-disease}. The three kinds of edges and their weights are described as miRNA similarity network, miRNA-disease network, and disease functional similarity network, respectively. For s ∈ ET and network ***A***_*s*_ ∈ {***RS***, ***RD***, ***DS***}, normalization is first implemented before further processing as follows:

(4) $$\begin{array}{r} {A_{s}}^{\prime }=\frac{A_{s}\left(i,j\right)}{\sum_{k=1}^{k=Col\left(A_{s}\right)}A_{s}\left(i,k\right)} \end{array}$$

where *Col*(***A***_*s*_) is the size of the ***A***_*s*_ column dimension and ***A***_*s*_(*i, j*) is the element in the *i*_*th*_ line and *j*_*th*_ column.

#### 2.4.2. Neighborhood Information Aggregation

To take full advantage of the heterogeneous network information, we adopted the neighborhood information aggregation strategy. First, an initial node embedding function ***f***:*N* → ***R***^*d*^ maps each node u to its d-dimensional vector representation ***f***(*u*). In our experiment, d is equal to 1024, and ***f*** is a function that outputs random values from a truncated normal distribution. Then, the neighborhood information aggregation can be defined as:

(5) $$\begin{array}{r} a_{u}=\underset{s\in ET}{\sum }\sum_{{A^{\prime }}_{s}\left(u,v\right)\neq 0}{A_{s}}^{\prime }\left(u,v\right)\cdot \sigma \left(f\left(v\right)\cdot W_{s}+b_{s}\right) \end{array}$$

where $W_{s}\in R^{d\times d}$ and $b_{s}\in R^{d}$ are the parameters trained in the neural network. In addition, σ(·) is the activation function in the neural network, and we used the RELU function here. Based on the graph convolutional operation, we pass the information of a node to its intermediate and distant neighbors and therefore realize the implicit influence among nodes on the network level.

#### 2.4.3. Updating the Node Embedding

Obtaining the aggregated neighbor information ***a***_*u*_, the process of updating the node embedding can be defined as:

(6) $$\begin{array}{r} f^{1}\left(u\right)=\frac{\sigma \left(W^{1}concat\left(f\left(u\right),a_{u}\right)+b^{1}\right)}{||\sigma \left(W^{1}concat\left(f\left(u\right),a_{u}\right)+b^{1}\right)|{|}_{2}} \end{array}$$

where ***f***^1^(*u*) is a new node embedding, ***W***^1^ ∈ ***R***^*d* × 2*d*^ is the weights, ***b***^1^ ∈ ***R***^*d*^ is the bias and || · ||_2_ is the *l*_2_ norm.

#### 2.4.4. Topology-Preserving Learning

Considering the same importance of preserving the known miRNA similarity (***RS***), disease similarity (***DS***) and miRNA-disease association (***RD***), we share all the parameters among these three subnetworks and minimize the loss function of reconstructing the entire heterogeneous network during the training process, as shown in Figure 1C. First, we use ***RF*** ∈ ***R***^*m* × *d*^ and ***DF*** ∈ ***R***^*n* × *d*^ to represent the feature matrix of miRNA and disease, respectively, where each row of the feature matrix represents a new node embedding ***f***^1^(*u*), m is the number of miRNA nodes, n is the number of disease nodes and d is the dimension of new node embedding. Then, topology-preserving learning of the node embedding can be defined as:

(7) $$\begin{array}{r} F=\left(\begin{array}{c} RF\\ DF \end{array}\right) \end{array}$$

(8) $$\begin{array}{r} min_{W^{1},b^{1},W_{s},b_{s},P,H}||G-{FPHF}^{T}|{|}_{2}^{2} \end{array}$$

where ***P*** ∈ ***R***^*d* × *k*^ and ***H*** ∈ ***R***^*k* × *d*^ are projection matrices used to extract the principle features from node representations, and G is the graph constructed in Equation (3). We set k to 512 in our experiment. The unknown parameters can be trained in an end-to-end manner by performing gradient descent to minimize the total squared reconstruction error. In the training phase, we iterate 2,000 epochs to establish the optimal parameters with the minimum error value.

### 2.5. Interaction Probability Between MiRNA and Disease

Finally, the predicted interaction probability between miRNAs and diseases can be obtained from the reconstructed heterogeneous network as follows:

(9) $$\begin{array}{r} \left(\begin{array}{cc} {RS}^{{}^{\prime }} & {RD}^{{}^{\prime }}\\ {DR}^{{}^{\prime }} & {DS}^{{}^{\prime }} \end{array}\right)=FPHF^{T} \end{array}$$

(10) $$\begin{array}{r} {RD}_{predicted}=\left({RD}^{\prime }+{DR{}^{\prime }}^{T}\right)/2 \end{array}$$

By comparing the reconstructed ***RD***_**predicted**_ and the original ***RD*** matrix, we can discover potential miRNA-disease associations. The prediction procedure is summarized in **Algorithm 1**. The code and data can be obtained online^1^.

**Table:** Algorithm 1: The proposed algorithm.

**Input:** MiRNA similarity network, ***RS***; Disease similarity network, ***DS***; MiRNA-disease association network, ***RD***;
**Output:** Predicted miRNA-disease associations, ***RD***_*predicted*_;
1: Construct a heterogeneous network ***G*** based on ***RS***, ***DS*** and ***RD***;
2: For each node u in graph ***G***, initialize its embedding as ***f***^0^(*u*);
3: Initialize parameters: $\theta^{1}=(W_{s}^{1}$, $b_{s}^{1}$, ***W***^1^, ***b***^1^, ***P***^1^, ***H***^1^);
4: *epochs* = 2000;
5: *minLoss* = *Inf*;
6: η = 0.0005;
7: *i* = 1;
8: **while** *i* <= *epochs* **do**
9: **for** each node u **do**
10: $a_{u}^{i}=\underset{s\in ET}{\sum }\underset{A_{s}^{{}^{\prime }}\left(u,v\right)\neq 0}{\sum }A_{s}^{{}^{\prime }}\left(u,v\right)\cdot \sigma \left(f^{i-1}\left(v\right)\cdot W_{s}^{i}+b_{s}^{i}\right)$;
11: $f^{i}\left(u\right)=\frac{\sigma \left(W^{i}concat\left(f^{i-1}\left(u\right),a_{u}^{i}\right)+b^{i}\right)}{||\sigma \left(W^{i}concat\left(f^{i-1}\left(u\right),a_{u}^{i}\right)+b^{i}\right)|{|}_{2}}$;
12: **end for**
13: ***RF***^*i*^ = (***f***^*i*^(1), ***f***^*i*^(2), ..., ***f***^*i*^(*m*))^*T*^;
14: ***DF***^*i*^ = (***f***^*i*^(*m* + 1), ***f***^*i*^(*m* + 2), ..., ***f***^*i*^(*m* + *n*))^*T*^;
15: $F^{i}=\left(\begin{array}{l} RF^{i}\\ DF^{i} \end{array}\right)$;
16: $\ell =||G-F^{i}P^{i}H^{i}{\left(F^{i}\right)}^{T}|{|}_{2}^{2}$;
17: **if** ℓ < *minLoss* **then**
18: *minLoss* = ℓ;
19: $\left(\begin{array}{ll} RS^{\prime } & RD^{\prime }\\ DR^{\prime } & DS^{\prime } \end{array}\right)=F^{i}P^{i}H^{i}{\left(F^{i}\right)}^{T}$;
20: ${RD}_{predicted}=\left({RD}^{{}^{\prime }}+{DR}^{{}^{\prime }T}\right)/2$;
21: **end if**
22: $\theta^{i+1}=\theta^{i}-\eta \nabla_{\theta^{i}}\ell \left(\theta^{i}\right)$;
23: *i* = *i* + 1;
24: **end while**
25: **return *RD***_***predicted***_;

### 2.6. Baseline Methods

We choose the following state-of-the-art methods as our baseline methods:

1. **MiRNA-disease association prediction based on matrix completion and label propagation (MCLPMDA)**: Yu et al. (2019) proposed a novel method named MCLPMDA. This method first reconstructs a similarity matrix of miRNA and disease by a matrix completion algorithm based on known experimentally verified miRNA-disease associations and then utilizes the label propagation algorithm to reliably predict potential disease-related miRNAs.
2. **Ensemble of decision tree based MiRNA-disease association prediction (EDTMDA)**: Chen et al. (2019) proposed a method named EDTMDA, which innovatively builds a computational framework integrating ensemble learning and dimensionality reduction.
3. **Predicting microRNA-disease associations based on sparse neighborhoods (SNMDA)**: Qu et al. (2018a) presented a method named SNMDA that takes advantage of the sparsity of the miRNA-disease association network and integrates the sparse information into the current similarity matrices for both miRNAs and diseases.
4. **MiRNA-disease association prediction based on global linear neighborhoods (GLNMDA)**: Yu et al. (2018) proposed a novel method that obtains a new miRNA/disease similarity matrix by linearly reconstructing each miRNA/disease according to the known experimentally verified miRNA-disease associations and then adopts label propagation to infer the potential associations between miRNAs and diseases.
5. **Predicting miRNA gene and disease relationship based on locality-constrained linear coding (LLCMDA)**: Qu et al. (2018b) proposed LLCMDA. This method first reconstructs similarity networks for both miRNAs and diseases using locality-constrained linear coding and then applies label propagation on the similarity networks to obtain relevant scores.
6. **Path-based computational model for miRNA-disease association prediction (PBMDA)**: You et al. (2017) constructed a heterogeneous graph consisting of three interlinked subgraphs and further adopted a depth-first search algorithm to infer potential miRNA-disease associations.
7. **Heterogeneous graph inference for miRNA-disease association prediction (HGIMDA)**: Chen et al. (2016) developed the computational model of HGIMDA to uncover potential miRNA-disease associations by integrating miRNA functional similarity, disease semantic similarity, Gaussian interaction profile kernel similarity, and experimentally verified miRNA-disease associations into a heterogeneous graph. HGIMDA adopts an iterative process to find the optimal solutions based on global network similarity information, which leads to superior performance over local network similarity-based methods.

## 3. Results

### 3.1. Performance Evaluation

Considering the uniqueness and limitedness of available miRNA and disease samples, we implemented LOOCV, LODOCV, and 5-fold cross-validation to evaluate the performance of our method (Jiao and Du, 2016). In each framework, we selected 5 state-of-the-art baseline models and plotted the receiver operating characteristic (ROC) curves of our method and the selected methods by calculating the false-positive rate (FPR) and true-positive rate (TPR) at varying thresholds.

LOOCV is conducted in two different ways: global and local LOOCV. In the framework of global LOOCV, one of the known miRNA-disease associations is left out in turn as a test sample, and the other known associations are regarded as training samples. All the unknown associations in the original ***RD*** matrix can be candidate samples. We ranked the predicted interaction scores of the test sample and the candidate samples. If the ranking of the test sample was higher than a threshold for a given true-positive rate (TPR), it was marked as positive. In the framework of local LOOCV, only the unknown associations of a specific disease are ranked with the test sample.

In 5-fold cross-validation, all the known miRNA-disease associations were randomly divided into five subsets. Each subset was taken as test samples in turn, and the others were considered training samples. All unknown miRNA-disease associations were considered candidate samples.

To further test the performance of our method in predicting associations for diseases without any known related miRNAs, we adopted LODOCV (Fu and Peng, 2017). In this framework, all the known miRNAs associated with a given disease were regarded as test samples.

**Figure 2:**
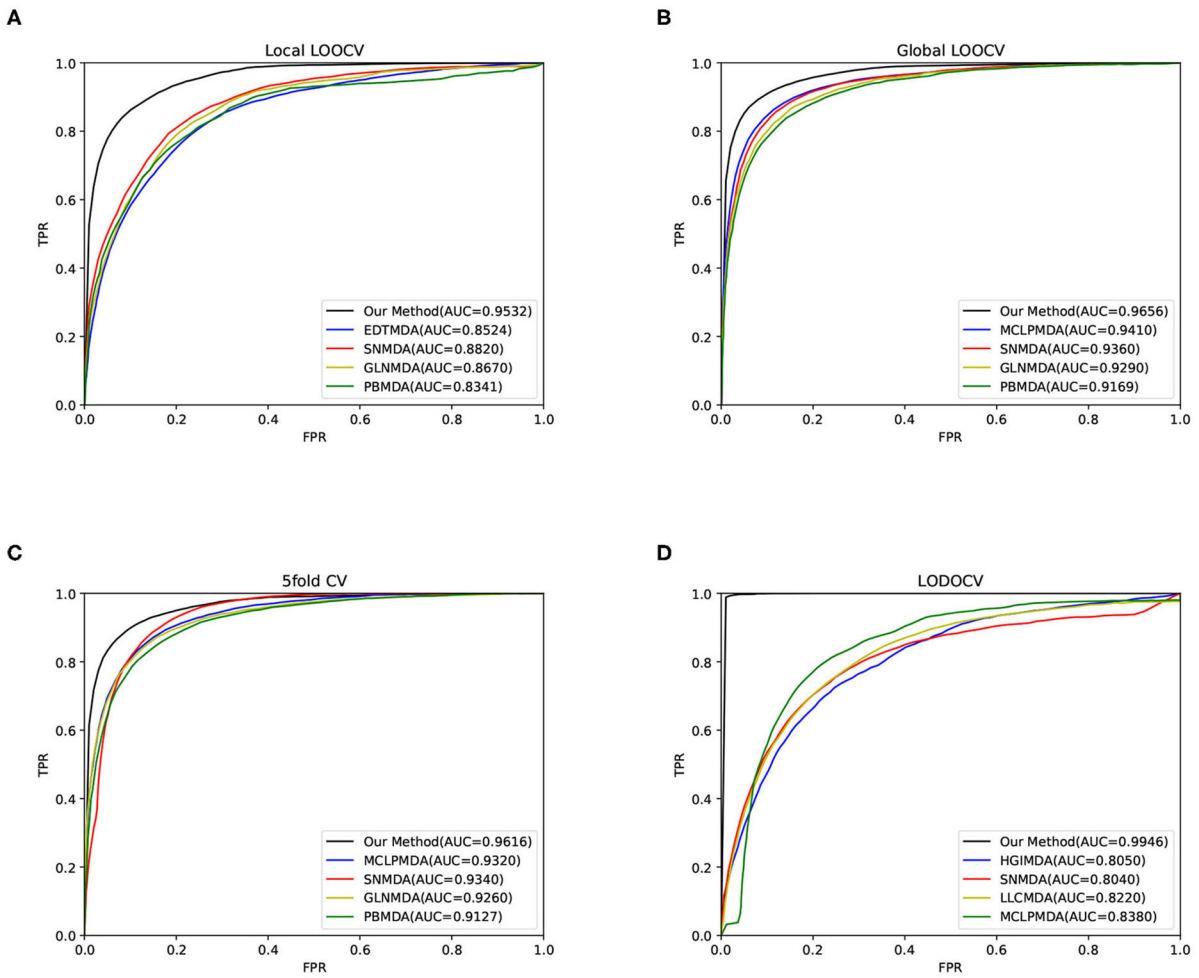
The results of our method and baseline methods in terms of **(A)** local LOOCV, **(B)** global LOOCV, **(C)** 5-fold cross-validation, and **(D)** LODOCV.

The area under the curve (AUC) was then calculated to evaluate the performance of our method. As a result, our method obtained AUCs of 0.9656, 0.9532, and 0.9616 in global LOOCV, local LOOCV, and 5-fold cross-validation, respectively, as shown in Figure 2. The performance of our method outperformed the baseline methods. For LODOCV, our method achieved the highest AUC value of 0.9946, which proved that our method could effectively predict new associations between miRNAs and diseases. We also note that the AUC value of LODOCV was much higher than that of LOOCV. The reason may be that the test samples of LODOCV are from the known miRNA-disease associations, the predicted interaction scores of which can be higher than those of the original unknown associations.

### 3.2. Case Studies

Two types of case studies were conducted to further validate the performance of the proposed method for novel miRNA-disease association prediction.

For the first type of case study, we applied the proposed method to predict novel miRNA-disease associations for three common human diseases (breast neoplasms, lung neoplasms, and prostate neoplasms) based on the known associations from HMDD. For a specific disease, known associations of all diseases were regarded as training samples, and unknown associations with this disease were regarded as candidate samples. After training the network, we ranked the prediction score of the candidate associations and selected the top 30 candidate associations with this disease. The prediction results were then verified by two databases: dbDEMC V2.0 (Yang et al., 2017) and PhenomiR (Ruepp et al., 2010). As a result, 28 out of the top 30 miRNAs were verified to be associated with breast neoplasms (Table 1), 27 out of the top 30 miRNAs were verified to be associated with lung neoplasms (Table 2), and 27 out of the top 30 miRNAs were verified to be associated with prostate neoplasms (Table 3). The results proved that our method can effectively predict potential miRNA-disease associations.

**Table 1:** The top 30 predicted miRNAs associated with breast neoplasms.

**miRNA(1-15)**	**Evidence**	**miRNA(16-30)**	**Evidence**
hsa-mir-30e	dbDEMC; PhenomiR	hsa-mir-192-2	Unconfirmed
hsa-mir-449a	dbDEMC; PhenomiR	hsa-mir-138-2	dbDEMC; PhenomiR
hsa-mir-15b	dbDEMC; PhenomiR	hsa-mir-142	dbDEMC; PhenomiR
hsa-mir-99b	dbDEMC; PhenomiR	hsa-mir-138-1	dbDEMC; PhenomiR
hsa-mir-542	dbDEMC	hsa-mir-29b	dbDEMC; PhenomiR
hsa-mir-98	dbDEMC; PhenomiR	hsa-mir-19b-2	dbDEMC; PhenomiR
hsa-mir-92b	dbDEMC	hsa-mir-185	dbDEMC; PhenomiR
hsa-mir-211	dbDEMC; PhenomiR	hsa-mir-32	dbDEMC; PhenomiR
hsa-mir-494	dbDEMC; PhenomiR	hsa-mir-130a	dbDEMC; PhenomiR
hsa-mir-150	dbDEMC; PhenomiR	hsa-mir-99a	dbDEMC; PhenomiR
hsa-mir-330	dbDEMC; PhenomiR	hsa-mir-186	dbDEMC; PhenomiR
hsa-mir-378a	dbDEMC; PhenomiR	hsa-mir-153-1	PhenomiR
hsa-mir-192	dbDEMC; PhenomiR	hsa-mir-451	dbDEMC; PhenomiR
hsa-mir-106a	dbDEMC; PhenomiR	hsa-mir-219-2	PhenomiR
hsa-mir-95	dbDEMC; PhenomiR	hsa-mir-128	Unconfirmed

**Table 2:** The top 30 predicted miRNAs associated with lung neoplasms.

**miRNA(1-15)**	**Evidence**	**miRNA(16-30)**	**Evidence**
hsa-mir-139	dbDEMC; PhenomiR	hsa-mir-708	dbDEMC
hsa-mir-92b	dbDEMC; PhenomiR	hsa-mir-429	dbDEMC
hsa-mir-19b-2	dbDEMC; PhenomiR	hsa-mir-192-2	unconfirmed
hsa-mir-152	dbDEMC; PhenomiR	hsa-mir-193b	dbDEMC; PhenomiR
hsa-mir-133a-2	dbDEMC; PhenomiR	hsa-mir-199a-2	dbDEMC; PhenomiR
hsa-mir-302b	dbDEMC; PhenomiR	hsa-mir-24-1	dbDEMC; PhenomiR
hsa-mir-378a	dbDEMC; PhenomiR	hsa-mir-625	dbDEMC
hsa-mir-125b-2	dbDEMC; PhenomiR	hsa-mir-451a	dbDEMC; PhenomiR
hsa-mir-10a	dbDEMC; PhenomiR	hsa-mir-451	dbDEMC; PhenomiR
hsa-mir-302c	dbDEMC; PhenomiR	hsa-mir-149	dbDEMC; PhenomiR
hsa-mir-130a	dbDEMC; PhenomiR	hsa-mir-151a	dbDEMC; PhenomiR
hsa-mir-106b	dbDEMC; PhenomiR	hsa-mir-128	unconfirmed
hsa-mir-125b	Unconfirmed	hsa-mir-128-1	dbDEMC; PhenomiR
hsa-mir-296	dbDEMC; PhenomiR	hsa-mir-219-2	PhenomiR
hsa-mir-345	dbDEMC; PhenomiR	hsa-mir-218-1	dbDEMC; PhenomiR

**Table 3:** The top 30 predicted miRNAs associated with prostate neoplasms.

**miRNA(1-15)**	**Evidence**	**miRNA(16-30)**	**Evidence**
hsa-mir-142	dbDEMC; PhenomiR	hsa-mir-196a-2	dbDEMC; PhenomiR
hsa-mir-9-3	dbDEMC; PhenomiR	hsa-let-7g	dbDEMC; PhenomiR
hsa-mir-9-1	dbDEMC; PhenomiR	hsa-mir-10b	dbDEMC; PhenomiR
hsa-let-7f-2	dbDEMC; PhenomiR	hsa-mir-429	Unconfirmed
hsa-mir-451a	dbDEMC	hsa-mir-196a-1	dbDEMC; PhenomiR
hsa-let-7f-1	dbDEMC; PhenomiR	hsa-mir-125b	Unconfirmed
hsa-mir-103a-1	dbDEMC; PhenomiR	hsa-mir-138-1	PhenomiR
hsa-mir-135a-2	dbDEMC; PhenomiR	hsa-mir-138-2	PhenomiR
hsa-mir-29b	unconfirmed	hsa-mir-210	dbDEMC; PhenomiR
hsa-mir-135a-1	dbDEMC; PhenomiR	hsa-mir-139	dbDEMC; PhenomiR
hsa-mir-7-1	dbDEMC; PhenomiR	hsa-mir-215	dbDEMC; PhenomiR
hsa-mir-103a-2	dbDEMC; PhenomiR	hsa-let-7a-2	dbDEMC; PhenomiR
hsa-mir-7-2	dbDEMC; PhenomiR	hsa-mir-181b-2	dbDEMC; PhenomiR
hsa-mir-7-3	dbDEMC; PhenomiR	hsa-let-7a-3	dbDEMC; PhenomiR
hsa-mir-199b	dbDEMC; PhenomiR	hsa-mir-218-2	dbDEMC; PhenomiR

In the second case study, we evaluated the ability of the proposed method to predict new associations for diseases without any known related miRNAs. We selected pancreatic neoplasms as an example in this case study. First, we set the known associations of pancreatic neoplasms as unknown associations, and all miRNAs were considered candidate miRNAs. Then, we implemented our method to obtain the prediction scores of these candidate miRNAs associated with pancreatic neoplasms. We found that 50 out of the top 50 miRNAs were confirmed by at least one database from dbDEMC v2.0 and Phe-nomiR v2.0 (Table 4). The results demonstrate that our method can be applied to predict potential associations for disease without any known related miRNAs.

**Table 4:** The top 50 predicted miRNAs associated with pancreatic neoplasms.

**miRNA(1-25)**	**Evidence**	**miRNA(25-50)**	**Evidence**
hsa-mir-133b	dbDEMC; PhenomiR	hsa-mir-10a	dbDEMC; PhenomiR
hsa-mir-103a-2	dbDEMC; PhenomiR	hsa-let-7d	dbDEMC; PhenomiR
hsa-mir-296	dbDEMC; PhenomiR	hsa-mir-100	dbDEMC; PhenomiR
hsa-mir-196a-1	dbDEMC; PhenomiR	hsa-let-7a-1	dbDEMC; PhenomiR
hsa-mir-143	dbDEMC; PhenomiR	hsa-mir-216a	dbDEMC; PhenomiR
hsa-mir-132	dbDEMC; PhenomiR	hsa-mir-425	dbDEMC; PhenomiR
hsa-mir-34b	dbDEMC; PhenomiR	hsa-mir-200b	dbDEMC; PhenomiR
hsa-mir-210	dbDEMC; PhenomiR	hsa-mir-224	dbDEMC; PhenomiR
hsa-mir-212	dbDEMC; PhenomiR	hsa-mir-99a	dbDEMC; PhenomiR
hsa-mir-26a-2	dbDEMC; PhenomiR	hsa-mir-128-2	dbDEMC; PhenomiR
hsa-mir-106a	dbDEMC; PhenomiR	hsa-let-7f-1	dbDEMC; PhenomiR
hsa-mir-26a-1	dbDEMC; PhenomiR	hsa-mir-183	dbDEMC; PhenomiR
hsa-mir-101-1	dbDEMC; PhenomiR	hsa-let-7f-2	dbDEMC; PhenomiR
hsa-let-7e	dbDEMC; PhenomiR	hsa-mir-135b	dbDEMC; PhenomiR
hsa-mir-451a	dbDEMC	hsa-mir-338	dbDEMC; PhenomiR
hsa-mir-25	dbDEMC; PhenomiR	hsa-let-7i	dbDEMC; PhenomiR
hsa-let-7b	dbDEMC; PhenomiR	hsa-mir-107	dbDEMC; PhenomiR
hsa-mir-200c	dbDEMC; PhenomiR	hsa-mir-10b	dbDEMC; PhenomiR
hsa-mir-27a	dbDEMC; PhenomiR	hsa-mir-191	dbDEMC; PhenomiR
hsa-let-7g	dbDEMC; PhenomiR	hsa-mir-186	dbDEMC; PhenomiR
hsa-mir-486	dbDEMC	hsa-mir-218-1	dbDEMC; PhenomiR
hsa-mir-101-2	dbDEMC; PhenomiR	hsa-mir-375	dbDEMC; PhenomiR
hsa-let-7a-2	dbDEMC; PhenomiR	hsa-mir-218-2	dbDEMC; PhenomiR
hsa-let-7a-3	dbDEMC; PhenomiR	hsa-mir-625	dbDEMC
hsa-mir-200a	dbDEMC; PhenomiR	hsa-mir-95	dbDEMC; PhenomiR

## 4. Discussion

In this paper, we propose a novel method to predict potential associations between miRNAs and diseases. The method constructs a heterogeneous network composed of the miRNA similarity network, disease similarity network, and known miRNA-disease association network. The miRNA similarity depends on the miRNAs and their possible families and clusters. The information of each node in this network is obtained by aggregating neighborhood information through graph convolutional networks. We compared the method with several state-of-the-art baseline methods. The method performed well in four types of cross-validations. Furthermore, two types of case studies were implemented. The results demonstrate that our proposed method is powerful in discovering potential disease-related miRNAs. In addition, the method can be used to predict the related miRNAs of diseases without any known association.

The reliable performance of the proposed method is due mainly to the following several important factors. First, we integrated useful datasets to construct a heterogeneous network. Second, the method made full use of the available information by aggregating neighborhood information derived from the heterogeneous network. Third, the parameters of the neural network were learned by minimizing the error of reconstructing the whole heterogeneous network, rather than that of just the miRNA-disease network.

However, there are still some limitations in our method. First, the datasets we used to construct the network possibly contain noise and outliers. Second, the heterogeneous network we constructed was insufficient to represent the complex relationships between miRNAs and diseases. Thus, our future research will focus on the diverse relationships between miRNAs and diseases.

## Data Availability Statement

All datasets generated for this study are included in the article/Supplementary Material.

## Author Contributions

RZ, YC, and HW conceived of the presented idea. RZ carried out the experiment and wrote the draft. CJ and YW helped shape the research, analysis, and manuscript. All authors discussed the results and contributed to the final manuscript.

## Conflict of Interest

The authors declare that the research was conducted in the absence of any commercial or financial relationships that could be construed as a potential conflict of interest.

## Footnotes

^1^https://github.com/aI-area/DMA
